# Supplementary material for: Internalization of Neutrophil-Derived Microvesicles Modulates TNFα-Stimulated Proinflammatory Cytokine Production in Human Fibroblast-Like Synoviocytes
Source: Int J Mol Sci. 2021 Jul 10;22(14):7409. doi: 10.3390/ijms22147409 (PMC8304992; doi:10.3390/ijms22147409)
Supplement: Supplementary file 1 [file ijms-22-07409-s001.zip › ijms-1268269 sup for production.pdf]

# Internalization of neutrophil-derived microvesicles modulates TNFα-stimulated proinflammatory cytokine production in human fibroblast-like synoviocytes

Dong Zhan, Andrew Cross, Helen L. Wright, Robert J. Moots, Steven W. Edwards, and Sittisak Honsawek

## Supplementary

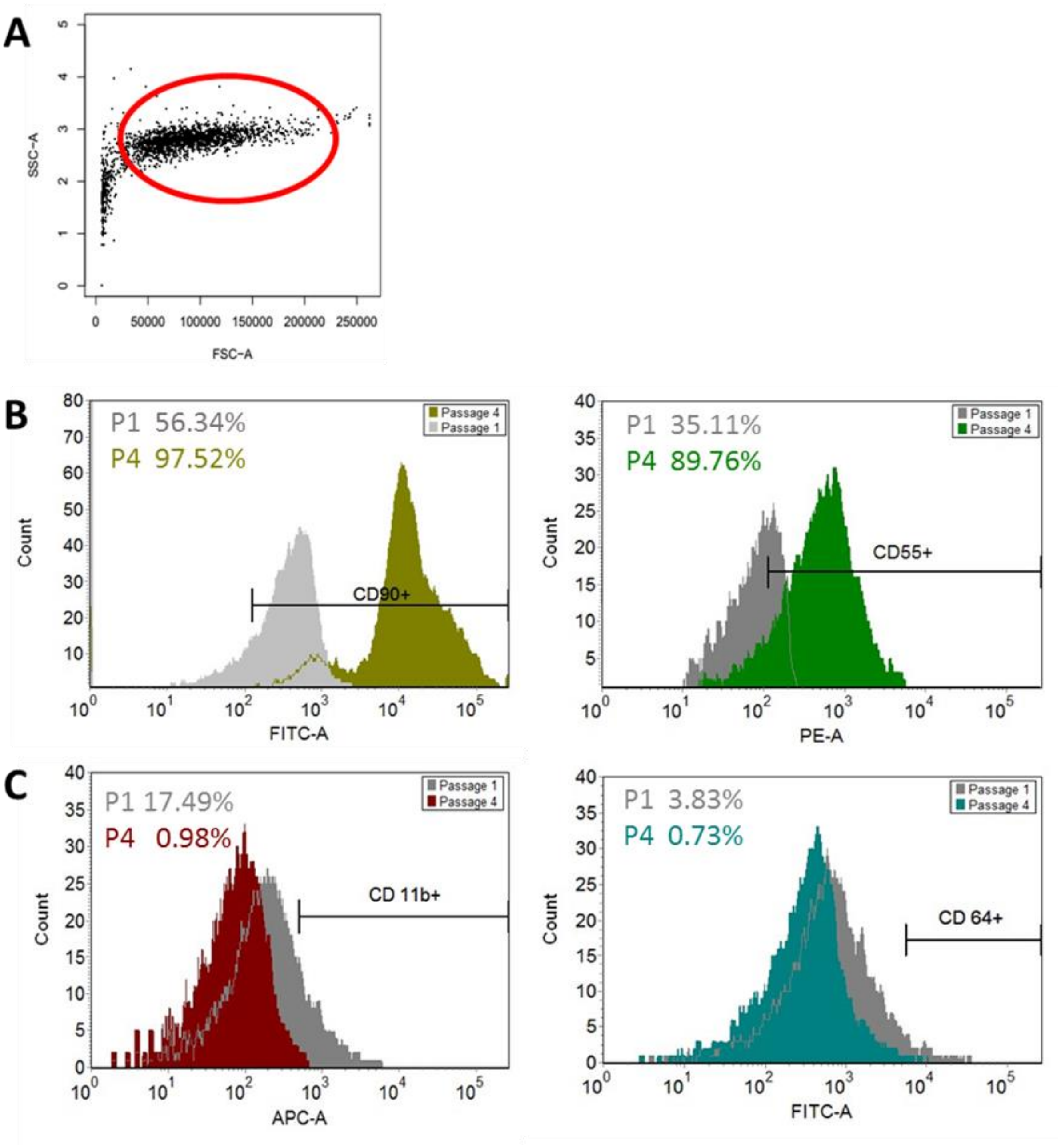


**Figure S1**. Flow cytometry to assess FLS purity. (A) scatter plot of FLS, gated by the red ellipse. (B) shows fibroblast positive CD markers and their percentage expression on P1 and P4 cells. (C) shows fibroblast negative CD markers and their percentage expression on P1 and P4 cells.

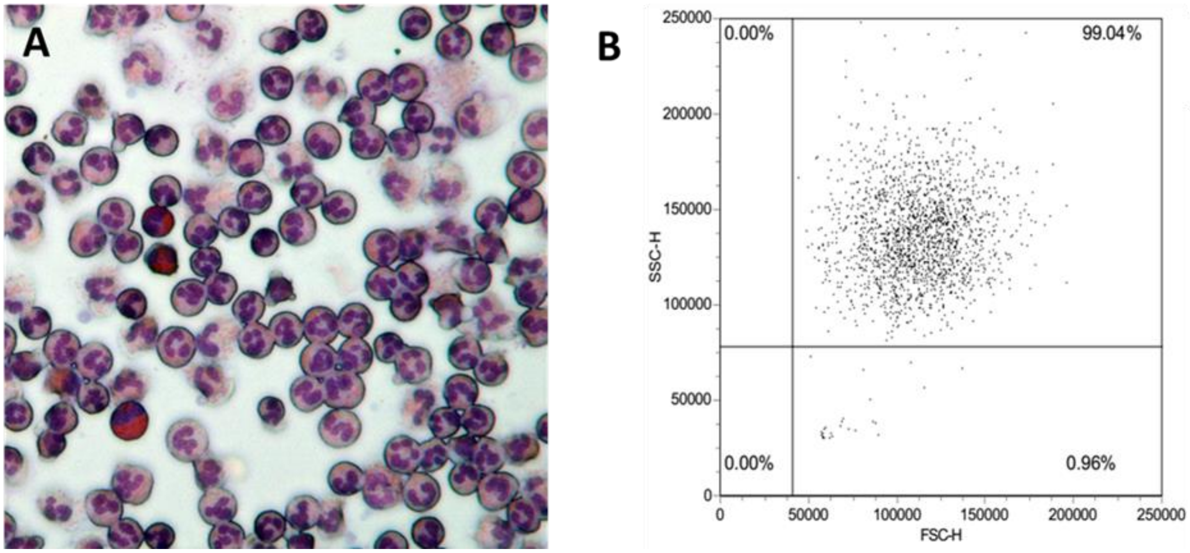


**Figure S2**. Isolated neutrophil purity by cytospin with Wright's staining in (A) and flow cytometry in (B).

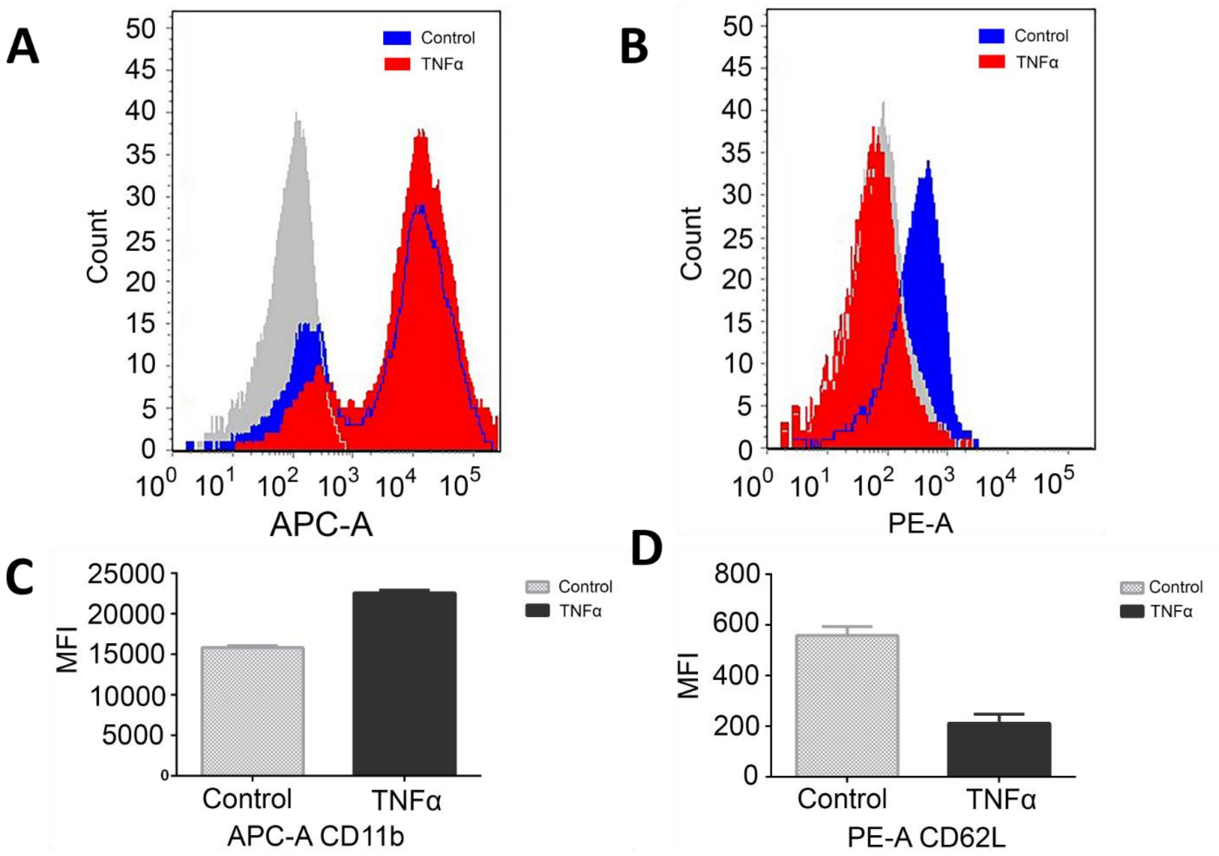


**Figure S3**. Isolated neutrophil functional examination: CD11b and CD62L expression on the surface of TNFα-treated and untreated neutrophils. (A) and (C) CD11b expression on neutrophils surface with/without treatment of 50 ng/mL TNFα for 20 min at 37℃. (B) and (D) CD62L expression on neutrophil surface. No significant difference was observed by Wilcoxon test. The MFI is shown as mean (±SE, n=3). MFI, mean fluorescence intensity.

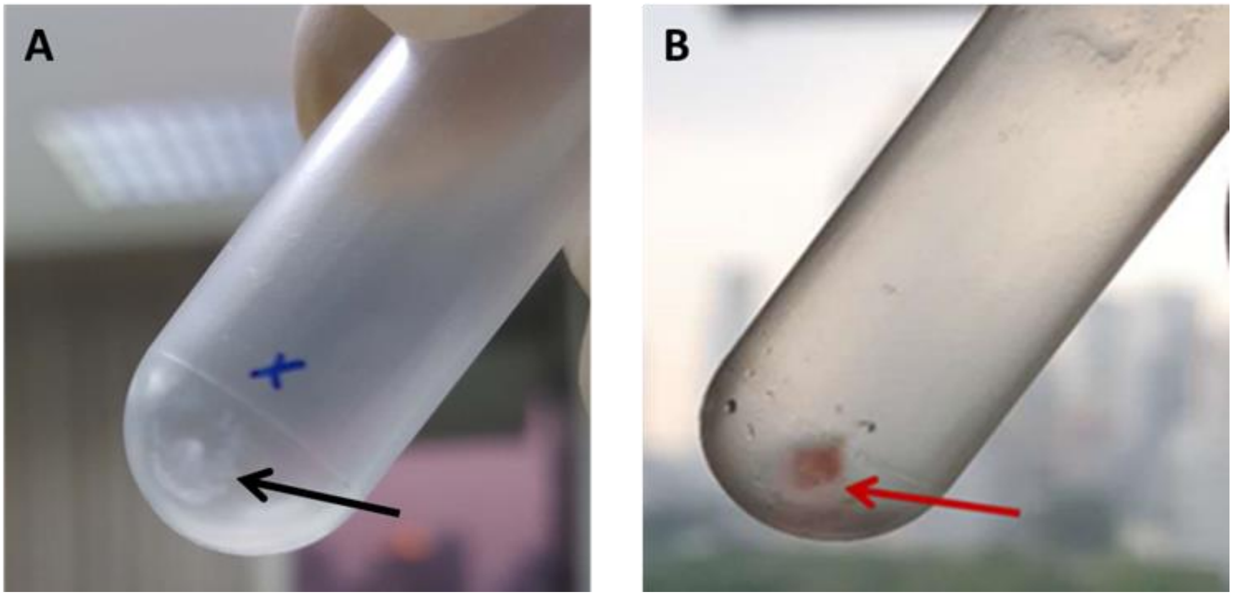


**Figure S4**. NDMVs pellet after ultra-centrifugation. (A) is unlabeled NDMVs and (B) is PKH26 labelled NDMVs.

**Supplementary Table S1.** Demographic data of knee OA patients in the present study.

| Patients | Age (years) | Gender (F/M) | BMI (kg/m$^2$) | KL score | VAS (0-10) | KOOS | ISOA | WOMAC |
|---|---|---|---|---|---|---|---|---|
| 1 | 72 | F | 20.08 | 4 | 5 | 25.00 | 17 | 105.00 |
| 2 | 69 | F | 26.64 | 3 | 3 | 25.00 | 15 | 115.59 |
| 3 | 65 | M | 29.05 | 3 | 6 | 43.75 | 10 | 253.82 |
| 4 | 58 | F | 25.10 | 2 | 5 | 31.25 | 11 | 208.53 |
| 5 | 64 | F | 27.06 | 2 | 3 | 12.50 | 15 | 168.68 |
| 6 | 61 | F | 24.09 | 2 | 3 | 18.75 | 14 | 148.97 |
| 7 | 73 | F | 23.11 | 2 | 4 | 18.75 | 14 | 155.44 |
| 8 | 77 | F | 22.06 | 3 | 9 | 12.50 | 13 | 202.06 |
| 9 | 77 | M | 21.33 | 4 | 3 | 12.50 | 17 | 52.21 |
| 10 | 65 | F | 25.24 | 2 | 8 | 12.50 | 14 | 121.62 |
| 11 | 73 | F | 27.34 | 2 | 7 | 12.50 | 13 | 160.88 |
| 12 | 81 | M | 22.77 | 2 | 7 | 37.50 | 11 | 155.29 |
| 13 | 67 | F | 25.68 | 2 | 4 | 37.50 | 14 | 201.62 |
| 14 | 81 | F | 32.65 | 2 | 8 | 37.50 | 13 | 155.00 |
| 15 | 72 | F | 31.20 | 2 | 5 | 25.00 | 8 | 147.21 |

Note: BMI: Body mass index, F: Female, M: Male, KL score: Kellgren and Lawrence grading system, KOOS: Knee injury and Osteoarthritis Outcome Score, ISOA: Indices of severity and disease activity for osteoarthritis, VAS: Visual analog scale, WOMAC: Western Ontario and McMaster Universities Arthritis Index.

**Supplementary Table S2.** Evaluation system of TUNEL immunohistochemistry (IHC) staining for FLS.

| | |
|---|---|
| Positive cells percent and evaluation | |
| Percent (%) | score |
| PC=0 | 0 |
| 0 < PC ≤ 25 | 1 |
| 25 < PC ≤ 50 | 2 |
| 50 < PC ≤ 75 | 3 |
| 75 < PC ≤ 100 | 4 |
| Positive cells intensity and evaluation | |
| Intensity | score |
| non (-) | 0 |
| weak (+) | 1 |
| mild (++) | 2 |
| strong (+++) | 3 |
| Total evaluation | |
| Total score of IHC = score of positive cells percent + score of positive cells intensity | |

**Supplementary Table S3.** The detectable ranges of 17 human cytokines levels in Bio-Plex Pro Human Cytokine 17-plex Assay.

| Cytokine | Detective range | |
|---|---|---|
| | Lower | Upper |
| IL-1β | 3.2 | 3,261 |
| IL-2 | 2.1 | 17,772 |
| IL-4 | 2.2 | 3,467 |
| IL-5 | 3.1 | 7,380 |
| IL-6 | 2.3 | 18,880 |
| IL-7 | 3.1 | 6,001 |
| IL-8 | 1.9 | 26,403 |
| IL-10 | 2.2 | 8,840 |
| IL-12 p70 | 3.3 | 13,099 |
| IL-13 | 3.7 | 3,137 |
| IL-17 | 4.9 | 12,235 |
| G-CSF | 2.4 | 11,565 |
| GM-CSF | 63.3 | 6,039 |
| IFN-γ | 92.6 | 52,719 |
| MCP-1 | 2.1 | 1,820 |
| MIP-1β | 2 | 1,726 |
| TNF-α | 5.8 | 95,484 |
